# Emergence and Cytogenetic Clonal Evolution of Chromosome 7 Abnormalities in Myeloid Malignancies: Investigating the Role of Telomere Dysfunction

**DOI:** 10.3390/ijms26031162

**Published:** 2025-01-29

**Authors:** Carmen Baldazzi, Lorenza Bandini, Valentina Robustelli, Agnese Patuelli, Claudia Venturi, Alessandra Grassi, Giulia Marzocchi, Angela Ielpo, Vincenza Solli, Maria Teresa Bochicchio, Stefania Paolini, Chiara Sartor, Federico Zingarelli, Antonio Curti, Emanuela Ottaviani, Nicoletta Testoni

**Affiliations:** 1 IRCCS Azienda Ospedaliero-Universitaria di Bologna, Istituto di Ematologia “Seràgnoli”, 40138 Bologna, Italy; 2 Dipartimento di Scienze Mediche e Chirurgiche, Università di Bologna, 40138 Bologna, Italy; 3 Biosciences Laboratory, IRCCS Istituto Romagnolo per lo Studio dei Tumori (IRST) “Dino Amadori”, 47014 Meldola, Italy

**Keywords:** myeloid neoplasm, clonal evolution, monosomy 7, deletion 7q, chromosomal instability, telomeres

## Abstract

Monosomy 7 and deletion 7q are common chromosomal abnormalities in myeloid malignancies, and they are associated with a poor prognosis. The mechanism underlying their acquisition remains elusive. We identified a cohort of 24 patients exhibiting clones with different chromosome 7 abnormalities, such as deletion 7q, unstable derivatives (ring chromosomes or ‘naked’ centromeres), and monosomy 7. We designated this group as having cytogenetic clonal evolution of chromosome 7 abnormalities (CCE7). In some cases, CCE7 correlated with disease progression, suggesting that deletions or other derivatives involving the q-arm of chromosome 7 may arise early in the disease course. These abnormalities may be transient but can potentially evolve into monosomy 7. Within the CCE7 group, telomere loss or shortening may contribute to chromosomal instability and the emergence of unstable derivatives, as the chromosome 7 derivatives displayed loss or rearrangement of subtelomeric regions. Moreover, we identified variants in genes implicated in telomere biology disorders and observed specific genetic mutation profiles associated with different chromosome 7 abnormalities. These findings shed light on a potential mechanism leading to monosomy 7 through the evolution of chromosome 7q abnormalities. Identifying patients at risk of developing monosomy 7, based on the presence of unstable derivatives with telomere loss or a specific mutation profile, could potentially enhance patient management and guide the development of novel therapeutic strategies.

## 1. Introduction

Monosomy 7 (-7) and partial deletion of the long arm of chromosome 7 (del(7q); 7q-) are frequent chromosomal abnormalities in acute myeloid leukemia (AML) [1] and myelodysplastic syndrome (MDS) [2]. Monosomy 7/del(7q) is frequently observed in patients previously exposed to cytotoxic therapy (i.e., alkylating agents) and/or radiotherapy [3].

In the most recent risk classification of AML and MDS, only monosomy 7 is included in the high-risk group [4,5]. Some studies highlighted that the prognosis of patients with -7 seems to be worse than those with del(7q) [6,7]. However, other studies did not confirm this difference [8,9,10]. These discrepancies may be due to the eventual presence of additional alterations; monosomy 7 often co-occurs with other alterations with an adverse prognosis such as MECOM rearrangements [11,12,13]. Additionally, deletions may differ in size and involve regions affecting the outcome [14,15]. Different data also exist regarding the genetic backgrounds of patients with del(7q) or -7; some studies suggest different mutation profiles while others report the same coexisting mutations [9,10].

Another elusive point is the mechanism underlying the genesis of -7/del(7q) and whether partial and complete loss of chromosome 7 share the same origin.

Studies on aplastic anemia (AA) showed an association between telomere shortening and progression to AML with the acquisition of monosomy 7, suggesting telomere dysfunction as a possible mechanism leading to -7 acquisition [16]. Chromosome 7 abnormalities are also the most frequently observed cytogenetic abnormalities during progression to MDS or AML in patients affected by inherited bone marrow failure syndromes (IBMFs). These hematological disorders are often characterized by telomere shortening caused by an accelerated cell turnover, strengthening the role of telomeres in the onset of monosomy 7 [17]. Furthermore, -7/del(7q) were the most frequently observed cytogenetic abnormalities detected in patients with telomere shortening syndromes that developed MDS or AML, which were the most common observed tumors [18]. Mutations in genes involved in telomeres maintenance are found in patients with IBMFs and telomere-shortening syndromes but also in patients with acquired AA [19,20,21].

In our study, cytogenetic analyses revealed that some patients exhibited the coexistence of clones with del(7q) and monosomy 7, supporting the hypothesis that del(7q) and -7 may represent different phases of clonal evolution triggered by chromosome 7 instability. A better understanding of the mechanisms leading to 7q abnormalities could help to develop new strategies to identify patients with a high risk of developing -7 clones and to improve therapeutic approaches.

We investigated the role of telomere and chromosome instability in the emergence of chromosome 7q abnormalities and further assessed the genetic background in patients with MDS or AML and different chromosome 7q abnormalities.

## 2. Results

### 2.1. Clinical and Cytogenetic Characteristics of Patients with Chromosome 7q Abnormalities (abn(7q))

Among 75 patients with MDS and AML exhibiting abn(7q) by chromosome banding analysis (CBA), we identified 21 with del(7q), 30 with -7, and 24 with a clone with -7 but also at least another clone with a different alteration involving chromosome 7q. These last 24 patients were characterized by either a clone with -7 and another clone with -7 and a marker chromosome, or a clone with -7 and at least one additional clone with del(7q) or other derivatives involving the 7q arm. The different clones were detected in the same or in sequential cytogenetic studies. We define these cases as patients with cytogenetic clonal evolution of chromosome 7 abnormalities (CCE7), as cytogenic clonal evolution can be diagnosed by sequential or by the parallel occurrence of metaphases at different evolution steps in a single cytogenetic analysis.

The diagnosis was AML in 44 cases (58.7%), MDS in 25 cases (33.3%), and MDS/AML with MDS gene-related mutation in 6 cases (8%). MDS diagnosis was more frequent among del(7q) cases, while AML was predominant in -7 and CCE7 cases. In 23 cases (30.7%), the disease was therapy-related, and in 15 cases (20%), it was secondary to previous MDS or myeloproliferative neoplasm (MPN), with a higher frequency in the CCE7 group. In 37 cases (49.3%), abn(7q) was found in the context of a complex karyotype (CK), while in 23 cases (30.7%), it was the sole abnormality. *MECOM* rearrangements were the most common recurrent abnormalities in patients with abn(7q) (13 cases) and were exclusively present in patients with -7 or CCE7. Clinical and biological data of the 75 patients are summarized in Table 1 and Supplementary Table S1.

### 2.2. Cytogenetic Clonal Evolution of abn(7q)

Regarding the cytogenetic characterization of patients with CCE7, most cases (18/24, 75%) exhibited two clones: one with -7 and another one with -7 plus an additional marker chromosome or del(7q).

**Figure 1:**
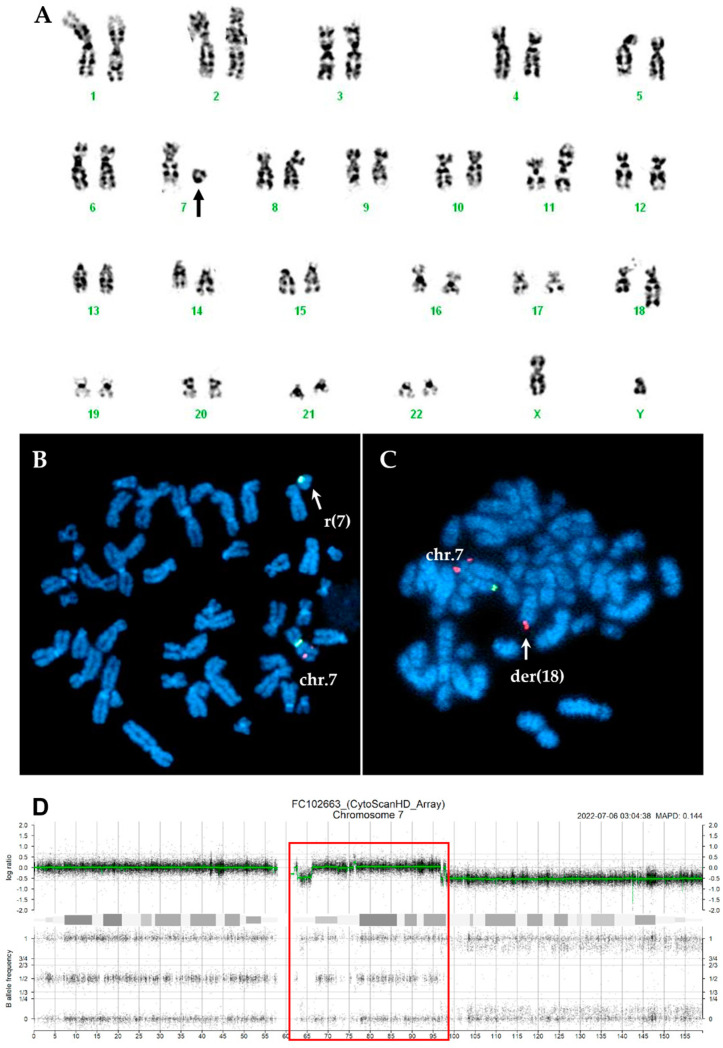
(**A**) Karyotype of case n.18 showing a r(7) indicated by the arrow. (**B**) The centromeric sequence of chromosome 7 (green signal) was detected on a marker chromosome resembling a ring by CBA. (**C**) Both the 7p and 7q-subtel probes were absent on the ring chromosome. The 7p-subtel probe (red signal) was translocated on the qter of der(18) involved in a complex translocation, whereas the 7q-subtel (green signal) was lost. (**D**) SNP-array profile of chromosome 7 showing the regions involved in ring formation (red box).

**Figure 2:**
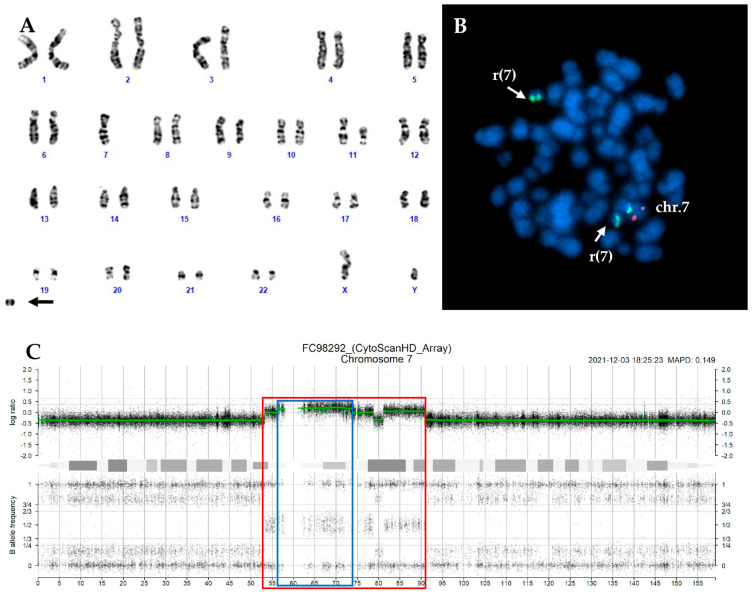
(**A**) Karyotype of case n.20 showing a r(7) indicated by the arrow. (**B**) The centromeric sequence of chromosome 7 (green signal) was detected on two marker chromosomes resembling a ring by CBA. (**C**) SNP-array profile of chromosome 7 showing the regions involved in ring formation (red box) and duplication (blue box) with multiple breakpoints and CN variation.

**Figure 3:**
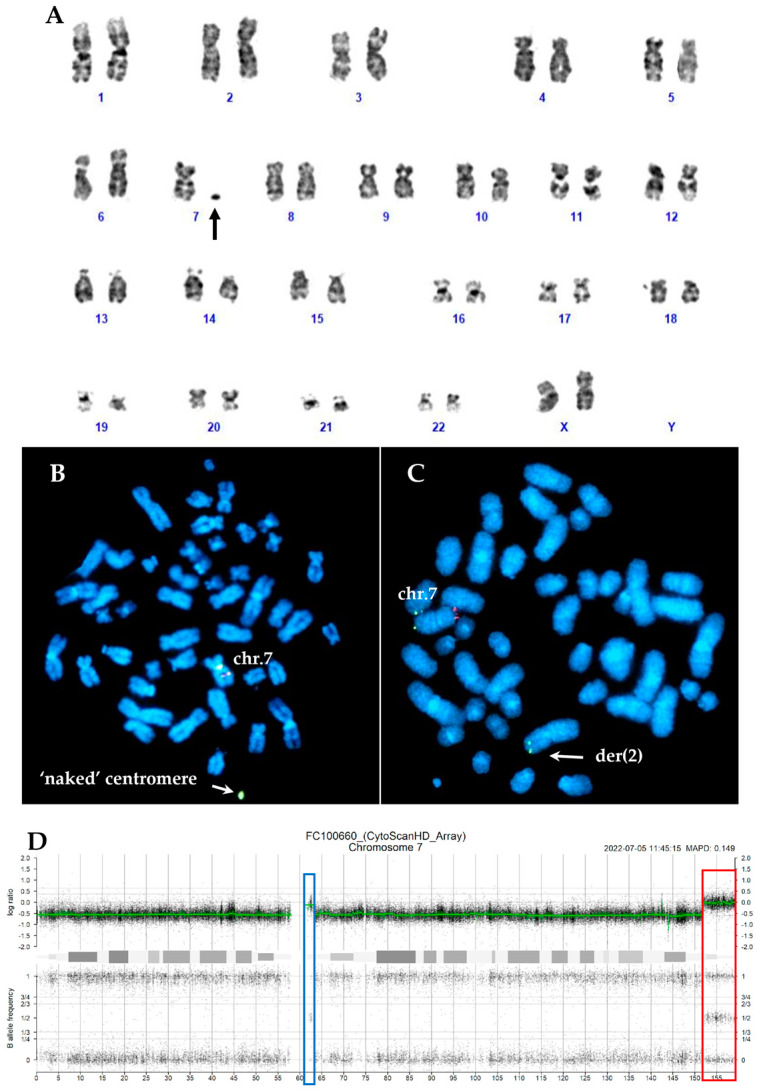
(**A**) Karyotype of case n.3 showing one ‘naked’ centromere indicated by the arrow.(**B**) The centromeric sequence of chromosome 7 was displayed on a ‘naked’ centromere (green signal). (**C**) The 7q-subtel probe (green signal) was detected on the pter of chromosome 2, whereas the 7p-subtel (red signal) was lost. (**D**) SNP-array profile of chromosome 7 showing the region translocated on der(2) (red box) and a small region involved in ‘naked centromere’ (blue box).

**Figure 4:**
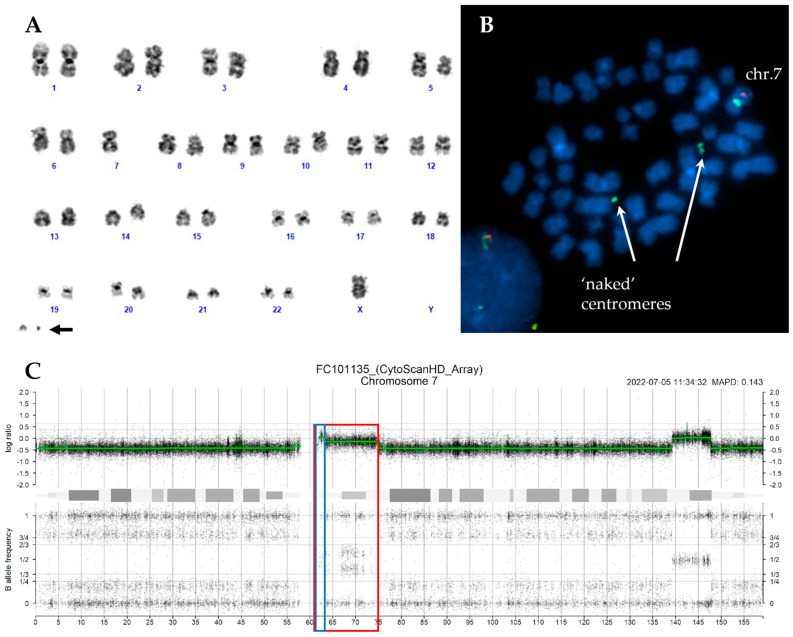
(**A**) Karyotype of case n.24 with duplication of ‘naked’ centromeres indicated by the arrow. (**B**) The centromeric sequence of chromosome 7 (green signal) was detected on two apparently ‘naked’ centromeres with different sizes. (**C**) SNP-array profile of chromosome 7 showing the regions probably involved in the two markers’ chromosome formation: one ‘naked’ centromere (red box) and one bigger ‘naked’ centromere (blue box).

In thirteen cases, the marker chromosomes were consistent with a ring chromosome (Figure 1A and Figure 2A), while in the remaining four cases, they were small chromosome fragments that appear to consist solely of centromeric sequences, referred to as ‘naked’ centromeres (Figure 3A and Figure 4A).

Six patients (6/24, 25%) (n.1–6, Table 2) had three clones, each with different abn(7q): one with deletion 7q, one with -7 plus a marker chromosome (either ‘naked’ centromeres, ring chromosomes or derivative 7q), and one with -7 alone. 

FISH analysis confirmed that all marker chromosomes derived from chromosome 7 as indicated by the presence of the centromere of chromosome 7 (Table 2).

In most cases, the different clones were observed in the same cytogenetic study (18/24, 75%), while in six cases (6/24, 25%) (n.1–3 and 10–12, Table 2), they emerged in sequential cytogenetic studies. In three cases, the del(7q) or the marker chromosome preceded the emergence of -7, which soon became the dominant clone. Notably, in these cases, CCE7 was associated with clinical progression from chronic myelomonocytic leukemia (CMML) to AML (Supplementary Figure S1). Two cases showed also a clone harbouring the duplication of the derivatives chromosome 7 (n.20, 24 Table 2).

Interphase FISH was performed using two different probes that overall marked the centromeric region and covered 7q22, 7q31, and 7q36 regions. In all CCE7 patients, at least two clones were detected: one with monosomy 7 and another with a 7q deletion. In two cases, an additional FISH pattern confirmed the duplication of the ring or ‘naked’ centromeres observed in CBA. In most cases (19/24, 79.2%), the deletion spanned all three analyzed regions (7q22, 7q31, and 7q36). FISH analysis with specific subtelomeric probes for chromosome 7 on metaphases with ring chromosomes and ‘naked’ centromeres revealed the absence of the telomeric regions on the derivatives chromosome 7. In most cases, both regions were lost, but in three cases one subtelomeric region was translocated to the end of other chromosomes (Table 1, Figure 1 and Figure 4).

SNP array analysis, performed in 16 cases with CCE7, allowed a more detailed characterization of the ring chromosomes. The deleted regions on ring chromosomes were highly variable in size and breakpoint localization; however, in most cases, the p-arm was almost completely lost, while the breakpoint on the q-arm was clustered in the 7q21 and 7q31 regions. In the case with duplication of the ring chromosome (n.20, Table 2), the SNP array confirmed the duplication of the region (arr[GRCh37] 7p11.2q11.23(56,679,152_73,517,086)x2~3) as well as a complex pattern with multiple breakpoints leading to ring formation and duplication with loss of sequences (Figure 2).

In four cases (n. 3,4,19 and 24, Table 2) with ‘naked’ centromeres, the SNP array highlighted complex rearrangements involving chromosome 7. In case n.3, which showed the 7qter region translocated to chromosome 2pter, SNPs confirmed the deletion of almost whole chromosome 7, except for two regions: a telomeric one arr[GRCh38] 7q35q36.3(148,149,167_156,685,399)x1~2 and pericentromeric one arr[GRCh38] 7q11.22q11.23(71,964,091_76,163,046)x1~2. No copy number variation (CNV) was observed at chromosome 2p, and the subtelomeric 2p probe was localized on chromosome 2pter, suggesting a 7qter-2pter telomere-telomere fusion (Figure 3).

In case n.24 (Table 2), one clone exhibited a ‘naked’ centromere, while another clone showed two ‘naked’ centromeres. SNP array analysis revealed the regions involved in the formation of the ‘naked’ centromeres, which differed in size and, considering the subclonality inferred from the copy number (CN) state and allelic imbalance, we hypothesized that one ‘naked’ centromere arises from the duplication of the other (Figure 4).

In two cases, more complex mechanisms such as chromothripsis or chromoanasyntesis could be suspected based on the SNP array profile (Supplementary Figure S2).

It is noteworthy that complex mechanisms involved in the acquisition of -7 were also identified in nine cases with -7 and complex karyotype but without evidence of CCE7 by CBA. In these cases, FISH and SNP array analyses highlighted the incomplete loss of chromosome 7 (Supplementary Figure S3). One case showed a chromosome 7 SNP profile resembling those with CCE7 (Supplementary Figure S3A). These data could suggest the presence of different clones that went undetected by CBA.

### 2.3. Identification of Variants in Genes Involved in Telomeres Maintenance

To assess the involvement of genes related to telomere maintenance, we tested 56 patients with abn(7q) (21 with del(7q), 15 with CCE7 and 20 with -7) and 14 patients without abn(7q) using a custom NGS panel of 22 telomere-related genes.

Results showed 30 heterozygous and 1 homozygous variant in 12 genes: *RTEL1* (n = 5), *PARN* (n = 4), *MDM4* (n = 4), *TERT* (n = 3), *NAF1* (n = 2), *WRAP53* (n = 2), *CTC1* (n = 2), *POT1* (n = 2), *TINF2* (n = 2), *ACD* (n = 2), *BLM* (n = 1), *TEN1* (n = 1), *ZCCH8* (n = 1) (Table 3, Supplementary Table S2).

Twenty-three patients with abn(7q) showed at least one variant (23/56, 41.1%) (range 1–3): 4 with CCE7 (4/15, 26.7%), ten with -7 (2 showed complex mechanism leading to -7) (10/20, 50%) and nine with del(7q) (9/21, 42.8%). Among the fourteen patients without abn(7q), four showed one variant (28.6%).

Although the limited number of cases, we observed that the most common variants in patients with abn(7) (*RTEL1*, *PARN*, *TERT*) were absent or less frequent (*MDM4*), than in patients without abn(7q). There was also a prevalence of specific mutations among the different abn(7q) groups: *MDM4* variants were more common in CCE7 cases and *TERT* variants in del(7q), whereas *PARN* and *RTEL* variants were observed more frequently in -7 patients.

Twenty-seven variants were classified as uncertain significance (VUS). Eight were likely benign (LB) and three benign (B). Nine out of 11 LB/B variants were classified as VUS or with conflicting interpretations in the Clinvar database or were previously reported in articles in association with telomere shortening (Supplementary Table S2).

Seven variants have not been reported; six were missense, one was a splicing variant, and one was a frameshift likely pathogenic variant in exon 2 of the *PARN* gene. The new frameshift *PARN* mutation (*PARN*: c.22_40del, p.(Phe8fs)) was observed in a young woman with a previous history of AA and normal karyotype; she progressed to MDS with the acquisition of monosomy 7 as the sole cytogenetic abnormality. Another splice variant of *PARN* was identified in the same patient. All *PARN* variants occurred in young patients associated with -7. Two novel missense *RTEL1* variants were identified in the same patient with -7 (Table 3).

### 2.4. Assessment of Telomeres Length

Telomeres length analysis was evaluated by quantitative FISH (Q-FISH) on interphase nuclei of 59 patients with abn(7): 18 with CCE7, 21 with 7q-, and 20 with -7. Additionally, Q-FISH was performed in seven cases with various trisomies. The different cytogenetic groups of patients were comparable in terms of median age and male/female ratio. Patients with abn(7q) showed longer average telomeres length compared to patients with trisomies (median T/C ratio: 4.09 and 2.80, respectively) (Supplementary Figure S4A).

**Figure 5:**
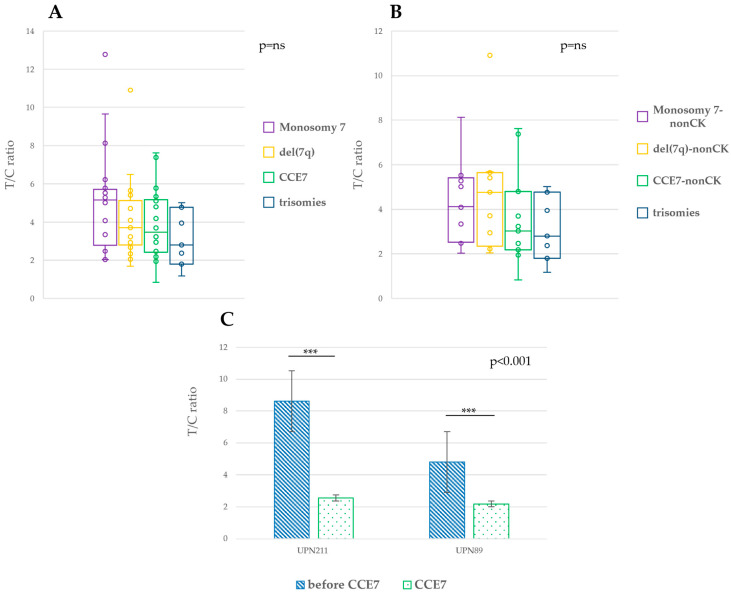
Telomere length analysis in patients with abn(7q) and various trisomies. (**A**) Average telomere length among CCE7, del(7q), -7, and trisomies groups of patients. (**B**) Average telomere length among CCE7, del(7q), and -7 patients with non-complex karyotype and trisomies. (**C**) Telomere length analysis in two CCE7 patients at the time of CCE7 emergence and 1 (UNP89) and 3 (UPN211) years before. ***: *p* < 0.001. ns: not significant.

Among patients with abn(7q), those with CCE7 showed shorter average telomeres length compared to del(7q) and -7 patients (median T/C ratio: 3.46, 3.81 and 5.19, respectively) (Figure 5A).

When considering only abn(7q) patients without complex karyotype (abn(7q)-nonCK), we observed the same trend; telomeres were longer in patients with abn(7q)-nonCK compared to those with trisomies (median T/C ratio: 3.90 and 2.80, respectively) (Supplementary Figure S4B). Among the different groups of abn(7q)-nonCK, CCE7 patients have shorter telomeres compared to those with del(7q) and -7 (median T/C ratio: 3.03, 4.76, and 4.11, respectively) (Figure 5B). However, the observed differences did not reach statistical significance. In two cases with CCE7, we measured the telomeres length before CCE7 emergence (1 and 3 years before) and determined that, at the time of CCE7, the average telomere lengths were much shorter than before. This suggests a rapid shortening of telomeres before CCE7 emergence (Figure 5C) and indicates a possible causative role in driving chromosome 7 instability.

### 2.5. Genomic Landscape of Patients with abn(7)

We next aimed to characterize the genetic background of patients with abn(7q) by analyzing the frequencies of additional mutations and cytogenetic abnormalities. In total, 110 mutations were identified in 26 of the 30 tested genes, with an average of 2 mutations per patient (range: 0–5). The most frequent mutated gene was *TP53* (22/56, 39.3%) followed by *DNMT3A* (11/56, 19.6%), *ASXL1* (7/56, 12.5%), *RUNX1* (7/56, 12.5%), *TET2* (6/56, 10.7%), and *PTPN11* (6/56, 10.7%), *EZH2* (5/56, 8.9%), and *U2AF1* (5/56, 8.9%) (Table 4, Supplementary Figure S5).

**Figure 6:**
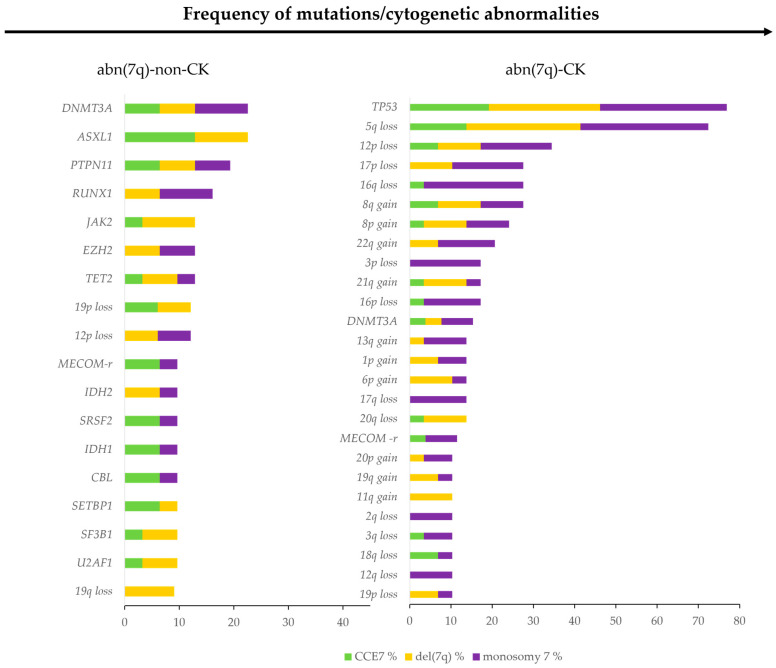
Co-existing somatic mutations and chromosomal aberrations in myeloid neoplasm with abn(7q). Percentage of co-associated mutation and cytogenetic abnormalities in abn(7)-non-CK (**left**) and abn(7q)-CK (**right**) in the three different groups of abn(7q).

Twenty-five patients (25/56, 44.6%) had a complex karyotype. In this sub-group, 40 mutations were identified, with 20 involving the *TP53* gene, which was the most frequently mutated gene (20/25, 80%) followed by *DNMT3A* (4/25, 16%). In 11 patients (11/25, 44%), *TP53* was the only mutated gene (Figure 6 and Supplementary Table S4). Five patients showed two heterozygous *TP53* mutations.

Thirty-one patients (31/56, 55.4%) did not have a complex karyotype. In this subgroup, 70 mutations were identified, with more than half of the patients (22/31, 70.9%) having at least 2 mutations. The most frequently mutated genes were *DNMT3A* (7/31, 22.6%), *ASXL1* (7/31, 22.6%), *PTPN11* (6/31, 19.2%), *RUNX1* (5/31, 16.1%), and *EZH2*, *TET2,* and *JAK2* (4/31, 12.9%) (Figure 6 and Supplementary Table S5).

Patients without CK karyotype had a significantly higher number of mutations per patient compared to CK patients, and among the abn(7q) group, CCE7 had the highest number of mutations per patient.

We identified specific mutational patterns for CCE7, del(7q), and -7 groups. CCE7 patients had higher frequencies of *ASXL1* (25%, *p* = 0.043), *CBL* (18.8%), and *ETV6* (12.5%) mutations, *RUNX1* mutations were more frequent in the -7 group (19%), while *U2AF1* mutations were more frequent in the del(7q) group (21.1%, *p* = 0.045). Genes involved in epigenetic regulation were the most frequently mutated in abn(7) patients (71.4%). Differences in mutation classes were also observed among the different groups.

Patients with -7 had less mutation in genes involved in chromatin modification than CCE7 and del(7q) groups (9.5%, 37.5%, and 36.8%, respectively). Mutations in genes involved in signal activation were more common in patients with CCE7 respect del(7q) and -7 patients (56.3%, 26.3%, and 23.8%, respectively), while mutations in genes involved in spliceosome complex were more frequently observed in del(7q) patients than CCE7 and especially -7 (47.4%, 25%, 19%, respectively). Mutations in the transcription factor class were more common in CCE7 (25%) and -7 (28.6%) and less frequently observed in del(7q) (5.3%) (Table 4 and Figure 6).

Based on molecular karyotyping, the most frequent CNVs were deletion 5q (37%), followed by deletion 12p (23%), deletion 17p (15%), deletion 16q (15%), trisomy 8 (15%), loss 19p and loss 16p (11%) (Figure 6).

Like with the mutation profile, we identified a specific association between some cytogenetic abnormalities and different abn(7q) groups. In particular, del(17p) was absent in the CCE7 patients compared to the del(7q) (15.8%) and -7 groups (23.1%). Loss 20q, loss 19q, and gain 11q were significantly more frequent in del(7q) (21.1%, 15.8%, and 15.8%, respectively), whereas loss 3p and loss 16q were strongly associated with -7 (15.4% and 30.8%, respectively) (Figure 6 and Supplementary Table S3).

## 3. Discussion

Monosomy 7 is a frequent aneuploidy in myeloid neoplasms and is associated with a poor prognosis [4]. While the precise mechanism leading to monosomy 7 remains unclear, it is generally hypothesized that aneuploidy arises from chromosome missegregation during mitosis in a single-step event [22,23].

In our study, we identified a cohort of patients with MDS or AML who exhibited different clones with diverse chromosome 7q abnormalities, including monosomy 7. Our findings suggest that the acquisition of monosomy 7 may also occur through a multi-step clonal evolution process. This process involves the formation of unstable chromosome 7 derivatives showing telomere loss and chromosomal instability. Indeed, most of the chromosome 7 derivatives observed in this subset of cases were ring chromosomes (69%), which are rare chromosomal abnormalities in hematologic malignancies [24]. Chromosome 7 is the most frequently involved chromosome in the formation of isolated ring chromosomes, and its presence is almost exclusively limited to the myeloid lineage [25], but, to our knowledge, its coexistence with monosomy 7 has never been reported. Ring formation can occur through breakage of the p and q chromosomal arms and fusion of the broken proximal ends, resulting in loss of distal material, or by telomere dysfunction, resulting in fusion of the reactive chromosomal ends [26]. Ring chromosomes are unstable, can undergo extensive changes in number and conformation [26,27], and can eventually be lost, producing monosomic cells [27].

In 16% of cases, we observed ‘naked’ centromeres, very small chromosome fragments, containing primarily repeated and conserved α-satellite DNA sequences that could explain their persistence. This finding has not been previously described in the literature. Telomeres on rings and ‘naked’ centromeres were lost (87.5%) or translocated to other chromosomes’ ends (12.5%). Telomere loss can trigger DNA repair mechanisms and telomere fusions, resulting in dicentric or ring chromosomes. Chromatin bridges are formed from chromosome end-to-end fusions, break, and can trigger break-fusion-bridging cycles (BFB cycles) when they are simultaneously pulled towards opposite poles during anaphase [26]. These cycles lead to multiple chromosomal rearrangements and deletions, including the formation of ring chromosomes and naked centromeres.

Naked centromeres were also associated with complex chromosomal rearrangements, such as chromothripsis, which is characterized by numerous clustered chromosomal rearrangements within limited genomic regions. Telomere shortening and subsequent BFB cycles are implicated in chromothripsis [28], as evidenced by the frequent occurrence of breakpoints within telomeric regions.

It is important to note that complex chromosomal rearrangements involving chromosome 7 were observed in a substantial proportion (30%) of patients with apparent monosomy 7 by conventional cytogenetics (CBA). Therefore, the true frequency of this multi-step clonal evolution process might be underestimated, as intermediate derivative chromosomes may be overlooked by CBA due to the growth advantage of monosomy 7 clones.

Several studies have emphasized the role of telomere shortening in inducing chromosomal instability [29,30]. While we did not observe significant differences in average telomere length between groups, CCE7 cases showed a trend towards shorter telomeres compared to those with deletions or monosomy 7. Furthermore, two cases exhibited telomere shortening before the emergence of chromosome 7 instability.

CCE7 is the early stage that precedes the acquisition and the expansion of the -7 clone; therefore, it is possible that, once monosomy 7 arises, telomeres could be stabilized and even increase in length due to telomerase reactivation or through alternative telomere lengthening (ALT) mechanisms [31]. Moreover, the shortest telomere within a cell, rather than the average length, maybe the critical determinant of chromosomal instability [32].

Previous chemotherapy or radiotherapy can also induce double-strand breaks (DSBs), leading to chromosome 7 extremity loss and instability without necessarily affecting average telomere length.

The role and the prevalence of genes involved in telomere biology disorders (TBD) in adults with hematologic malignancies are not well characterized. Most studies focused on familial cases with hematological disorders [33]. In these cases, *TERT*, *TERC,* and *RTEL1* variants were the most frequently described in AML or MDS patients, while heterozygous *PARN* variant carriers were associated with blood count abnormalities [33]. To the best of our knowledge, there are no studies that addressed the association with specific cytogenetic subgroups.

In our study, mutational analysis in genes involved in telomere maintenance or TBD showed a higher frequency of potential clinically relevant variants in patients with abn(7q) compared to patients without abn7q. The most frequently affected genes were *PARN, RTEL, TERT,* and *MDM4* and were prevalent in patients with abn(7q).

*PARN* variants were observed in three young patients with -7: one with MDS, one with MDS/AML, and one with AML. It is noteworthy that a young woman developed MDS following AA treated with immunosuppressants and showed two variants in the *PARN* gene: one was a new likely pathogenetic frameshift variant. Heterozygous *PARN* variant carriers were reported in association with blood count abnormalities but without hematological malignancies [33].

Defective telomere homeostasis is implicated in a significant group of patients with AA and patients with lower telomere content had a higher risk for clonal evolution and for acquisition of monosomy 7 compared to patients with higher telomere content [34]. 

*RTEL1* variants were identified in five patients (four AML and one MDS), three showed -7, and two had del(7q). One patient with -7 showed a biallelic *RTEL1* variant. Heterozygous variants in *RTEL1*, which encodes a protein involved in TERC processing and critical for telomere homeostasis, were also observed in hematological malignancies. Interestingly, some RTEL1 variants were not associated with significant telomere shortening but with excessive 3′ overhang erosion and inappropriate telomere capping [35].

*MDM4* variants were found in five patients: three with CCE7, of which two harbored the same variant (MDM4:c.1120A>C): one with -7 and one patient with normal karyotype. All five patients were diagnosed with AML. *MDM4* is well known for its role as an essential and specific negative regulator of p53 [36]. Recently, a germline missense mutation in the *MDM4* coding region has been described in a family that exhibited some dyskeratosis congenita (DC)-like phenotypic traits [37]. The authors used a mouse model to show that this mutation led to activation of p53, short telomeres, and bone marrow failure [37]. The same authors had previously reported that mice expressing an overactive p53 mutant lacking its C terminal recapitulated the complete phenotype of DC patients [38]. Five independent patients with a germline mutation in *MDM4* were reported in two additional studies: all presented bone marrow abnormalities, and two exhibited very short telomeres, confirming the impact of *MDM4* on telomere maintenance [39,40,41,42]. Thus, although alterations in the p53/MDM2/MDM4 regulatory node are known to promote cancer, these studies seem to demonstrate that mutations affecting this pathway may be associated with telomere shortening syndrome phenotypes.

Genetic characterization of patients with abn(7q) showed some common genetic background but also specific mutational profiles associated with different abn(7q). *TP53, DNMT3A, ASXL1, PTPN11, TET2,* and *RUNX1* were the most frequently mutated genes, which is consistent with previous studies [8,9,10,13,14].

The first major difference in the genetic profile of patients with abn(7q) was found between CK and non-CK. The former exhibited a high number of copy number variations (CNVs) and a limited number of mutations, with *TP53* being the most frequent mutation and the only mutated gene in about 40% of cases. The association between *TP53* and complex karyotype has already been widely reported in the literature [43]. In contrast, cases without CK had a higher number and diversity of mutations, predominantly affecting *DNMT3A*, *PTPN11*, and *ASXL1*.

The most frequent CNVs included losses on chromosomes 5q, 12p, 17p, 16p, 16q, and 19p, and gains on chromosomes 8q and 8p corresponding to trisomy 8. The association between abn(7q) and loss 5q, loss 12p, loss 17p, and gain 8q has already been established [44], while the association with loss 16q, loss 16p, and loss 19p has not been previously described.

Our analysis revealed distinct mutational and cytogenetic landscapes among different chromosome 7 abnormalities. While some alterations, such as losses on 5q and 12p, and mutations in *TP53* and *DNMT3A*, were common across all groups, others showed specific associations.

In the CCE7 group, we observed a significantly higher frequency of *ASXL1* mutations in genes involved in activated signaling pathways compared to the monosomy 7 group. Notably, *ASXL1* is one of the most common mutated genes in AA [21]. Loss 20q, loss 19q, gain 11q, and *U2AF1* mutations were specifically associated with del(7q), while loss 3p and loss 16q were associated with -7 cases, which also showed a higher frequency of *RUNX1* mutations compared to the other groups.

In conclusion, we identified a distinct group of patients who develop monosomy 7 through a complex mechanism involving clonal evolution and unstable derivatives.

While average telomere length assessments did not reveal significant differences, several observations, such as telomere loss, subtelomeric rearrangements, and telomere shortening preceding the emergence of chromosome 7 abnormalities, suggest a correlation between telomere deficiency and CCE7 and could play a role in driving this process. Monosomy 7 is challenging to treat with stem cell transplant, being the only effective modality [45]. The identification of unstable derivatives of chromosome 7 with telomere loss may indicate an increased risk of evolving towards monosomy 7. In some patients, CCE7 was associated with clinical progression toward AML, highlighting its significance as an early stage in disease development. Identifying patients at risk of developing monosomy 7 and recognizing early signs of telomere dysfunction could address early intervention and guide therapeutic strategies.

Further research is essential to elucidate the mechanisms underlying the emergence and clonal evolution of chromosome 7 abnormalities, investigate the associated pathogenic pathways, and further explore the role of telomere dysfunction in this process.

## 4. Materials and Methods

### 4.1. Patients

This study was conducted on 75 adult patients with MDS or AML and abnormalities of the q arm of chromosome 7 (abn(7q)) by chromosome banding analysis (CBA) and referred to the “Seràgnoli” Institute of Hematology—IRCCS Azienda Ospedaliero-Universitaria di Bologna. Informed consent was obtained following the Declaration of Helsinki, according to protocols approved by the institutional ethics committee (414/2019/Sper/AOUBo). Conventional karyotyping, molecular cytogenetic characterization, and genetic analyses were performed at diagnosis and during the course of the disease.

### 4.2. Chromosome Banding Analysis (CBA)

CBA was performed on bone marrow (BM) cells after short-term culture (24 and/or 48 h). The cells were treated with colchicine and hypotonic solution. The pellet was fixed and washed in methanol–acetic acid (3:1). The cells were suspended in fixative and dropped on slides. Karyotypes were examined after G-banding techniques and described according to the International System for Human Cytogenetic Nomenclature (ISCN 2020) [46].

### 4.3. Fluorescent In Situ Hybridization (FISH)

FISH analysis was performed on interphase nuclei and metaphase spreads obtained using the CBA technique and stored at −20 °C, according to the manufacturer’s instructions. All patients were tested with D7S486/CEP7 FISH Probe Kit (Vysis Inc., Downers Grove, IL, USA), XL 7q22/7q36 Deletion Probe (MetaSystems Probes GmbH, Altlussheim, Germany), and 7p-arm and 7q-arm Subtelomere Specific Probes (Cytocell, Cambridge, UK).

The slides were counterstained with DAPI and analyzed using fluorescent microscopes equipped with FITC/TRITC/AQUA/DAPI filter sets (Nikon Instruments, Tokyo, Japan) and the Genikon imaging system software version 7.22.3 (Nikon Instruments). At least 200 interphase nuclei were analyzed for each sample.

### 4.4. Quantitative Fluorescent In Situ Hybridization (Q-FISH)

Q-FISH was performed on interphase nuclei from unstimulated BM cells after 24 or 48 h’ culture using a Telomere PNA FISH Cy3 (HLB Panagene Co., Deajeon, Republic of Korea) specific for telomere sequence and according to the manufacturer’s instruction with slight modifications. Additionally, a FISH probe specific for the centromere of chromosome 2 (Agilent, Santa Clara, CA, USA) was used as internal control. 

Briefly, slides were immersed in 2Xsaline sodium citrate (SSC) buffer for 2 min, then dehydrated in a cold ethanol series (70%, 85%, and 100%) for 2 min each. After drying, a mixture of 1 µL of PNA cen(2) FITC probe and 9 µL of PNA telomere Cy3 probe was added. The slides were cover-slipped and sealed with rubber cement, then placed in pre-heated Hybrite adjusted to 80 °C for 10 min and hybridized at 37 °C overnight. Slides were washed at 60 °C for 10 min in 2XSSC with 0.1% Igepal and then for 2 min at room temperature in 0.4XSSC with 0.05% Igepal. The slides were air-dried, counterstained with DAPI, and finally analyzed using fluorescent microscopes equipped with FITC/TRITC/AQUA/DAPI filter sets (Nikon Instruments).

The digitized fluorescent telomere FISH signals were quantified using the open source, JAVA-based image analysis software ImageJ and a custom-designed plugin called “Telometer” version 3.1.0, URL (accessed on 12 October 2022) https://demarzolab.pathology.jhmi.edu/telometer/.

Telomere length (TL) was assessed on each cell, as the ratio of the total intensity of telomeric signals in each cell to the total intensity of centromeric signals in the same cell (T/C). TL was expressed as T/C. At least 100 nuclei were scored for each sample.

### 4.5. Next Generation Sequencing Analysis

Mutational status characterization of 22 genes directly and indirectly connected to telomere maintenance pathways and known to be involved in telomere biology disorders pathogenesis (reported in bold) [22] was performed using a customized NGS panel. The targeted gene panel was designed using the AmpliSeq Custom DNA Panel (Illumina, San Diego, CA, USA) and included ***TERC***, ***TERT***, ***DKC1***, ***TINF2***, ***RTEL1***, ACD, ***NOP10***, ***NHP2***, ***PARN***, ***WRAP53***, ***CTC1***, ***STN1***, ***NAF1***, *TERF1*, *TERF2*, *TERF2IP*, *TEN1*, *GAR1*, ***POT1***, *BLM*, ***MDM4***, and ***ZCCHC8***.

The custom panel was designed to obtain amplicons on all coding regions and exon-intron junctions (±25 base pairs) of the genes of interest. Total DNA was extracted from mononuclear cells isolated by Ficoll-Hypaque from patients’ bone marrow/peripheral blood collected at the time of abn(7q) emergence. Sequencing libraries were prepared according to the protocol provided by the manufacturer and sequenced with a paired-end protocol on the Illumina MiSeq platform, using 600-cycle V3 cartridges. Human Genome Build 19 (Hg19/GRC37) served as the reference for sequence alignment. FASTQ sequencing files were analyzed on Illumina’s Basespace Sequence Hub platform via the DNA Amplicon application. We obtained an average coverage of approximately 1000×in 99.89% of the target sequences, which consisted of 489 amplicons. 

The mutational analysis of 30 genes involved in myeloid leukemogenesis was conducted using The Myeloid Solution (SOPHiA Genetics, Saint Sulpice, Switzerland) NGS panel. This panel included the following genes: *ABL1* (exons 4–9), *ASXL1* (exons 9,11,12,14), *BRAF* (exon 15), *CALR* (exon 9), *CBL* (exons 8,9), *CEBPA* (all), *CSF3R* (all), *DNMT3A* (all), *ETV6* (all), *EZH2* (all), *FLT3* (exons 13–15,20), *HRAS* (exons 2,3), *IDH1* (exon 4), *IDH2* (exon 4), *JAK2* (all), *KIT* (exons 2,8–11,13,17,18), *KRAS* (exon 2,3), *MPL* (exon 10), *NPM1* (exon 10,11), *NRAS* (exons 2,3), *PTPN11* (exon 3,7–13), *RUNX1* (all), *SETBP1* (exon 4), *SF3B1* (exons10–16), *SRSF2* (exon 1), *TET2* (all), *TP53* (exons 2–11), *U2AF1* (exons 2,6), *WT1* (exons 6–10), and *ZRSR2* (all). Libraries were prepared according to the manufacturer’s instructions and paired-end sequenced (2 × 301) with 500-cycle V2 or 600-cycle V3 cartridges on a MiSeq™ instrument (Illumina, Inc., San Diego, CA, USA), following the manufacturer’s protocol. FASTQ sequencing files were analyzed by the SOPHiA DDM^®^ platform (SOPHiA Genetics, Saint Sulpice, Switzerland). Human Genome Build 19 (Hg19/GRC37) was used as a reference for sequence alignment.

Intronic and synonymous variants were filtered, reporting only those with a minor allele frequency (MAF) < 0.01, according to population databases (ExAC, 1000 Genomes, gnomAD v.2.1.1), and with a variant allele frequency (VAF) ≥ 0.02 for the myeloid panel and ≥0.05 for the custom panel. For variant classification and interpretation, Franklin Genoox v.76 (URL (accessed on 1 October 2024) https://franklin.genoox.com) and databases such as ClinVar (release: 9 September 2024) and COSMIC version.100, as well as in silico functional predictors (SIFT version 4.9, PolyPhen-2), were consulted.

### 4.6. SNP Array Analysis

DNA samples were processed using the Affymetrix^®^ (Santa Clara, CA, USA) genome-wide human CytoscanHD Array, following the manufacturer’s instructions. The .CEL files were analyzed with the R Rawcopy package (version 3.0) [47]. Subsequently, the copy number (CN) profile was corrected for ploidy using the R package BoBafit version 1.10.0 [48], which employs a list of chromosomes that typically do not undergo alterations in the disease to verify the proper centering of the diploid region. This list of normal chromosomes was created using the ComputeNormalChromosome package function, with a tolerance rate of 0.20. Only those chromosomes that were altered in the series used in the study with a frequency of less than 20% were taken as references. The default values of the function (<1.60 and >2.40) were applied to identify the altered chromosomal arms. Furthermore, the SNP array profile of chromosome 7 was rechecked against the CN profile plots of each sample using the ChAS (Chromosome Analysis Suite) software version 4.3.0.71 (Affymetrix).

### 4.7. Statistical Analysis

Statistical analysis of the study results was performed using STATA software version 18. The parametric Student’s *t* test, the non-parametric Mann–Whitney U test, and the Kruskal–Wallis test (for comparison of three or more groups) were used to compare continuous variables. The χ^2^ test for sufficiently large samples and the Fisher exact test for small samples (with fewer than five variables in at least one group) were employed to analyze the differences observed between nominal or ordinal categorical variables. For all statistical analyses, *p*-values ≤ 0.05 were considered significant.

## Figures and Tables

**Table 1:** Clinical and biological features of 75 patients with abn(7q).

	All n = 75	del(7q) n = 21	-7 n = 30	CCE7 n = 24
**Age, median**	66	68	62	66
**Sex (M/F)**	44/31	10/10 ^1^	19/11	15/10
**WBC/mm^3^, median**	2945	2535	3175	3125
**MDS, all n. (%)**	25(33.3)	10(47.6)	6(30)	9(37.5)
**AML all n. (%)**	44(58.7)	8(38.1)	20(66.7)	16(66.7)
**MDS/AML n. (%)**	6(8)	2(9.5)	4(13.3)	0
**Therapy-related n. (%)**	23(30.7)	6(28.6)	7(23.3)	10(41.7)
**AML secondary to previous MN ^1^ n. (%)**	15(20)	3(14.3)	4(13.3)	8(33.3)
**Complex karyotype n. (%)**	37(49.3)	10(47.6)	16(53.3)	11(45.8)
**Isolated abn(7) n (%)**	23(30.7)	6(28.6)	8(26.7)	9(37.5)
**MECOM rearrangement n. (%)**	13(17.3)	0	7(23.3)	6(25)

^1^ MN, Myeloid Neoplasm.

**Table 2:** Clinical and cytogenetic characteristics of 24 patients with CCE7.

Case	ID	Age/Sex	Pathology	Disease’s Phase	Karyotype	7p-Subtelomeric Region §	7q-Subtelomeric Region §
1	212	52/M	CMML	Diagnosis	46,XY,**del(7)(q22q36).ish del(7)(D7Z1+,D7S486-)[3]**/46,XY[17]		loss
				Follow-up	46,XY,**del(7)(q22q36)[4]**/46,XY,**add(7)(q22).ish add(7)(D7Z1+,D7S486-)[2]**/46,XY[14]		loss
				Progression	46,XY,**del(7)(q22q36)[8]**/45,XY,**-7[3]**/46,XY[12]		
		53/M	s-AML	Diagnosis	45,XY,**-7[18]**/46,XY[2]		
2	223	59/F	AML	Diagnosis	46,XX,del(5)(q13q33),**del(7)(q?)[1]** *		
				Follow-up	46,XX,del(5)(q13q33),**del(7)(q22).ish del(7)(D7Z1+,D7S486)**,del(8)(q22),del(9)(q22),der(10),der(12),+8,der(13)t(1;13)(p13;q14),der(16), mar1,+mar2[cp28]		on 12qtel
				Follow-up	47-48,XX,del(5)(q13q33),**add(7)(q22).ish add(7)(D7Z1+,D7S486-)**,del(8)(q22),del(9)(q22),der(10),der(12),der(17)+mar1+mar2[cp8]		on 12qtel
				Follow-up	43-45,XX,t(3;6)(p21;p23),del(5)(q13q33),**-7**,add(7)(p22),+8,i(11)(q10),-13,der(15)t(1;15)(p13;q15),-15,der(16), -19,+ mar[cp19]	loss	on 12qtel
3	219	56/F	AML	Diagnosis	46,XX,inv(3)(q21q26),-7,**+mar.ish der(7)(D7Z1+,D7S486-)[18]**/46,XX,inv(3)(q21q26), **del(7)(q22).ish del(7)(D7Z1+,D7S486-)[2]**	loss	on 2ptel
				Follow-up/Refractory	46,XX,inv(3)(q21q26),**-7**,**+der(7**)[9]/45,XX,inv(3)(q21q26),**-7[9]**	loss	on 2ptel
				Follow-up/Refractory	45,XX,inv(3)(q21q26),**-7[15]**/46,XX,inv(3)(q21q26),**-7,+der(7)[5]**	loss	on 2ptel
4	56	74/F	t-AML	Diagnosis	43,XX,del(5)(q13q33),**-7**,del(12)(p11p13),-16,-18[6]/43,XX,del(5)(q3q33),**-7**,del(12),-16,-18, **+mar.ish der(7)(D7Z1+,KMT2E-,D7S486-,EZH2-)[2]**/43,XX,del(5)(q13q33),**der(7)(q?).ish der(7)(D7Z1+,KMT2E-,D7S486-,EZH2-)**,del(12)(p11p13),-13,-18[2]	loss	loss
5	208	63/M	MDS	Diagnosis	45,XY,**-7[2]**/46,XY,**-7**,**+r(7).ish r(7)(D7Z1+,KMT2E-,D7S486-,EZH2-)[11]**/46,XY,**del(7)(q22q34).ish del(7)(D7Z1+,KMT2E-,D7S486-,EZH2+)[2]**	loss	loss
6	214	67/F	AML	Diagnosis	44,XY,der(5),-6,**-7**,+dmin[9]/45,XY,der(5),-6,**-7**,**+r(7).ish r(7)(D7Z1+,D7S486-)**,+dmin[5]/44,XY,der(5), -6,**der(7).ish der(7)(D7Z1+,D7S486-)**,del(4)(q27q33),der(17)add(17)(q25)[3]	loss	loss
7	89	66/M	CMML	Diagnosis	46,XY[20]		
		69/M	CMML	Progression	45,XY,**-7[11]**/46,XY,**del(7)(q22q36).ish del(7)(D7Z1+,D7S486-)[5]**/46,XY[14]		loss
			s-AML	Diagnosis	45,XY,**-7[20]**		
8	213	59/F	s-AML	Diagnosis	45,XX,**-7[26]**/46,XX,**-7**,**+r(7).ish r(7)(D7Z1+,D7S486-)[4]**	loss	loss
9	215	70/F	t-AML	Diagnosis	46,XX,**-7**,**+r(7).ish r(7)(D7Z1+,D7S486-)[16]**/45,XX,-7[6]	loss	loss
10	211	64/F	CMML	Diagnosis	46,XX[30]		
		67/F	CMML	Progression	46,XX,**-7**,**+r(7).ish r(7)(D7Z1+,D7S486-)[9]**/46,XX[21]	loss	loss
			s-AML	Diagnosis	46,XX,**-7**,**+r(7)[10]**/45,XX,-7[7]/46,XX[13]	loss	loss
11	220	48/M	s-AML	Diagnosis	45,XY,inv(3)(q21q26),**-7[12]**/46,XY,inv(3)(q21q26),**-7**,**+r(7).ish r(7)(D7Z1+,D7S486-)[11]**/46,XY[7]	loss	loss
				Relapse	45,XY,inv(3)(q21q26),**-7[19]**/46XY[1]		
12	222	49/M	AML	Diagnosi	45,XY,inv(3)(q21q26),**-7[15]**		
				Partial cytological response	45,XY,inv(3)(q21q26),**-7[12]**/46,XY,inv(3)(q21q26),**-7**,**+r(7).ish r(7)(D7Z1+,D7S486-)[4]**/46,XY[4]	loss	loss
13	216	58/M	t-AML	Diagnosis	45,XY,**-7[12]**/46,XX,**-7**,**+r(7).ish r(7)(D7Z1+,D7S486-)[3]**/46,XY[5]	loss	loss
				Follow-up	45,XY,**-7[15]**		
14	221	47/F	t-AML	Diagnosis	46,XX,**-7**,der(13)t(3;13)(q21;q34),**+r(7).ish r(7)(D7Z1+,D7S486-)[17]**/46,XX,**-7**,**+r(7**)[2]/45,XX,**-7**,der(13)(t(3;13)(q21;q34)[2] #	loss	loss
15	224	32/M	AML	Diagnosis	45,XY,t(3;14)(q21;q24),**-7[21]**/46,XY,t(3;14)(q21;q24),**-7**,**+r(7).ish r(7)(D7Z1+,D7S486-)[9]** ‡	loss	loss
				Follow-up/refractory	46,XY,t(3;14)(q21;q24),**-7**,**+r(7)[6]**/46,XY[24]	loss	loss
				Cytological remission	46,XY,t(3;14)(q21;q24),**-7**,**+r(7)[2]**/46,XY[28]	loss	loss
16	227	68/M	t-MDS	Diagnosis	45,X,-Y(4)/44,X,-Y,del(5)(q15q31),**-7**,-12,**+r(7).ish r(7)(D7Z1+,D7S486-)[6]**/45,X,-Y,del(5)(q13),**-7**,del(12)(p12p13),**+r(7).ish r(7)(D7Z1+,D7S486-)[2]**/44,X,-Y,dic(1;15)(p26p42;p11),del(5)(q13),**-7**,del(12)(p12p13)[6]	loss	loss
17	226	79/M	MDS	Diagnosis	45,XY,del(5)(q31q35),**-7**,del(11)(q12q24),-20,-21,**+r(7).ish r(7)(D7Z1+,D7S486-),**+1dmin[18]/45,XY,del(5)(q31q35),**-7**,del(11)(q12q24),-20,-21,+1dmin [2]		loss
18	217	66/M	t-AML	Diagnosis	45,XY,inv(3)(q21q26),t(5;18)(q31;q23),**-7[10]**/45,XY,t(5;18)(q31;q23),**-7**,der(11)t(11;11)(p15;q13)[6]/46,XY,t(5;18)(q31;q23),**-7**,**+r(7).ish r(7)(D7Z1+,D7S486-),**der(11)t(11;11)(p15;q13),[4]	on derivative chromosome 18	loss
19	225	70/M	t-AML	Diagnosis	44,XY,**-7**,hsr(11)(q13q23),-13,-17,del(20)(q11q13),-21,der(22)add(22)(p13),+mar1,**+mar2.ish der(7)(D7Z1+,D7S486-)[19]**/43,XY,**-7**,hsr(11)(q13q23),-13,-17,del(20)(q11q13),-21,der(22)add(22)(p13),+mar1[5]	on 9qtel	loss
20	129	54/M	s-AML	Diagnosis	46,XY,**-7**,del(11)(q13q23),**+mar.ish der(7)(D7Z1+,KMT2E-,D7S486-,EZH2-)[5]**/45,XY,**-7**,del(11)(q13q23)[3]/50,XY,+8,+8,del(11)(q13q23),**+mar.ish der(7)(D7Z1+,KMT2E-,D7S486-,EZH2-)x2[3]**	loss	loss
21	190	69/F	t-MDS	Diagnosis	46,XX,-7**,+r(7).ish r(7)(D7Z1+,KMT2E+,D7S486+,EZH2-)[5]**/45,XX,-7	loss	loss
22	193	69/F	AML	Diagnosis	45,XX,**-7[4]**/46,XX,**-7**,**+r(7).ish r(7)(D7Z1+,KMT2E+,D7S486+,EZH2-)[4]**	loss	loss
23	201	59/M	t-MDS	Diagnosis	46,XY,**-7**,**r(7).ish r(7)(D7Z1+,KMT2E-,D7S486-,EZH2-)[9]**/45,XY,**-7[3]**	loss	loss
24	203	81/F	s-AML	Diagnosis	45,X,-X,del(5)(q22q35),**-7**,**+mar.ish der(7)(D7Z1+,KMT2E-,D7S486-,EZH2-)[12]**/46,X,-X,del(5),**-7**,**+mar.ish der(7)(D7Z1+,KMT2E-,D7S486-,EZH2-)x2[8]**/45,X,-X,del(5)(q22q35),**-7**,+8,add(14)(p13),-18,**+mar.ish der(7)(D7Z1+,KMT2E-,D7S486-,EZH2-)[2]**/43,X,-X,del(3)(q13q27),del(5)(q22q35),**-7**,add(14)(p13),-18,del(20)(q11q13)[8]	loss	loss

Chromosome 7 abnormalities are evidenced in bold. CMML, chronic myelomonocytic leukemia, s-AML, secondary AML, t-AML, AML therapy related. * By FISH analysis, deletion 7q was detected in 3% on analyzed nuclei. # FISH with GATA2/MECOM probes showed three copy of MECOM gene. ‡ FISH with GATA2/MECOM probes showed complex GATA2/MECOM rearrangements. § FISH with specific subtelomeric probes. Only abnormal results or localization of the signals are reported.

**Table 3:** Clinical and genetic characteristics of patients with variants in genes involved in telomere biology related disorders.

ID	Age/Sex	Pathology	Abn(7q)	Telomere Gene Panel (VAF%)
16	26/F	MDS ^a^	-7	PARN:c.22_40del p.(Phe8fs) (51.3%); PARN:c.21T>C (splice variant) (52.6%)
113	55/F	MDS/AML	-7	PARN:c.1297C>A p.(Leu433Ile) (47.1%); CTC1:c.25C>T p.(Pro9Ser) (23.7%)
202	70/M	s-AML	-7	RTEL1:c.3392C>G p.(Thr1131Arg) (57.6%)
162	50/F	AML	-7	RTEL1:c.2544_2546delTGGinsCGA p.(Gly849Asp) (32.4%)
83	67/M	t-MDS/AML	-7	BLM:c.3062A>G p.(Asn1021Ser) (48.1%)
112	76/M	t-AML	-7	RTEL1:c.3354G>C p.(Met1118Ile) (52.0%); RTEL1:c.2544_2546delTGGinsCGA p.(Gly849Asp) (47.8%)
35	60/F	MDS/AML	-7	NAF1:c.208G>C p.(Val70Leu) (49.8%)
96	55/M	AML	-7	TINF2:c.734C>A p.(Ser245Tyr) (49.9%)
41	28/M	AML	-7 *	PARN:c.1785T>G p.(Asp595Glu(49.9%); MDM4:c.1162C>G p.(Pro388Ala) (50.6%)
207	70/F	t-AML	-7 *	TEN1:c.262G>A p.(Val88Met) (52.5%); TINF2:c.1213G>A p.(Glu405Lys) (49.9%)
193	78/F	s-AML	CCE7	MDM4:c.1120A>C p.(Lys374Gln) (47.9%)
219	56/F	AML	CCE7	MDM4:c.1100C>T p.(Ser367Leu) (47.5%)
215	74/F	t-AML	CCE7	CTC1:c.2386-10A>G (splice variant) (100%)
129	55/M	s-AML	CCE7	WRAP53:c.668G>C p.(Gly223Ala) (12%); MDM4:c.1120A>C p.(Lys374Gln) (52.0%); TERT:c.3184G>A p.(Ala1062Thr) (51.9%)
184	66/M	s-AML	del(7q)	POT1:c.1127A>G p.(Gln376Arg) (21.5%)
6	79/F	AML	del(7q)	TERT:c.2545A>C p.(Met849Leu) (50.3%)
22	62/F	MDS	del(7q)	WRAP53:c.305C>T p.(Thr102Ile) (46.7%);
130	62/M	t-AML	del(7q)	RTEL1:c.2624G>A p.(Arg875Lys) (51.4%)
12	35/M	MDS	del(7q)	RTEL1:c.3592G>A p.(Glu1198Lys) (50.7%)
186	75/M	MDS	del(7q)	ACD:c.22G>A p.(Val8Ile) (47.5%); TERT:c.3184G>A p.(Ala1062Thr) (47.5%)
11	64/F	MDS	del(7q)	TERT:c.1234C>T p.(His412Tyr) (50.2%)
81	70/M	MDS	del(7q)	NAF1:c.1478A>C p.(Tyr493Ser) (41.6%)
34	47/F	AML	del(7q)	POT1:c.1228G>C p.Asp410His (70.3%)
200	35/M	AML	No	MDM4:c.1153C>G p.(Leu385Val) (52%)
183	67/F	AML	No	ZCCHC8:c.1325C>T p.(Ala442Val) (46.0%)
181	31/F	AML	No	ACD:c.22G>A p.(Val8Ile) (48.9%); NAF1:c.879-7C>G (49.1%)
108	66/M	AML	No	ACD:c.243-6_243-4dup

^a^ previous Aplastic Anemia; VAF variant allele frequency; * -7 by CBA, FISH, and SNP array analyses revealed partial deletion of chromosome 7 with complex rearrangements.

**Table 4:** Comparison of gene alterations, grouped by functional categories, among patients with different abn(7q).

	All (n = 56)	CCE7 (n = 16)	del (7q) (n = 19)	-7 (n = 21)	*p* Value
**Epigenetics modifiers**	40 (71.4)	13 (81.3)	16 (84.2)	11 (52.4)	0.257
**DNA methylation**	25 (44.6)	7 (43.8)	9 (47.4)	9 (42.9)	0.968
*DNMT3A*	11 (19.6)	3 (18.8)	3 (15.8)	5 (23.8)	0.441
*TET2*	6 (10.7)	1 (6.3)	3 (15.8)	2 (9.5)	0.754
*IDH1*	4 (7.1)	2 (12.5)	1 (5.3)	1 (4.8)	0.666
*IDH2*	3 (5.4)	0	2 (10.5)	1 (4.8)	0.621
*WT1*	1 (1.8)	1 (6.3)	0	0	0.268
**Chromatin modifiers**	15 (26.8)	6 (37.5)	7 (36.8)	2 (9.5)	**0.072**
*ASXL1*	7 (12.5)	4 (25.0)	3 (15.8)	0	**0.043**
*EZH2*	5 (8.9)	0	3 (15.8)	2 (9.5)	0.302
*SETBP1*	3 (5.4)	2 (12.5)	1 (5.3)	0	0.185
**Activated signaling**	19 (33.9)	9 (56.3)	5 (26.3)	5 (23.8)	**0.082**
*FLT3-ITD*	1 (1.8)	1 (6.3)	0	0	0.286
*KRAS*	2 (3.6)	1 (6.3)	0	1 (4.8)	0.741
*NRAS*	1 (1.8)	0	0	1 (4.8)	>0.999
*PTPN11*	6 (10.7)	2 (12.5)	2 (10.5)	2 (9.5)	>0.999
*JAK2*	4 (7.1)	1 (6.3)	3 (15.8)	0	0.135
*CSFR3*	1 (1.8)	1 (6.3)	0	0	0.286
*CBL*	4 (7.1)	3 (18.8)	0	1 (4.8)	**0.093**
**Transcription factors**	12 (21.4)	4 (25)	1 (5.3)	6 (28.6)	0.154
*RUNX1*	7 (12.5)	1 (6.3)	1 (5.3)	4 (19.0)	0.365
*CEBPA*	3 (5.4)	1 (6.3)	0	2 (9.5)	0.496
*ETV6*	2 (3.6)	2 (12.5)	0	0	**0.078**
**Spliceosome-complex**	17 (30.4)	4 (25.0)	9 (47.4)	4 (19.0)	0.146
*U2AF1*	5 (8.9)	1 (6.3)	4 (21.1)	0	**0.045**
*SRSF2*	4 (7.1)	2 (12.5)	0	2 (9.5)	0.373
*ZRSR2*	4 (7.1)	0	3 (15.8)	1 (4.8)	0.190
*SF3B1*	4 (7.1)	1 (6.3)	2 (10.5)	1 (4.8)	0.670
**Tumor suppressor**	22 (39.3)	5 (31.3)	9 (47.4)	8 (38.1)	0.657
*TP53*	22 (39.3)	5 (31.3)	9 (47.4)	8 (38.1)	0.657

Data are presented as n (%). *p* values of ≤0.05 are statistically significant.

## Data Availability

The original contributions presented in this study are included in the article/Supplementary Material. Further inquiries can be directed to the corresponding author.

## Supplementary Materials

The following supporting information can be downloaded at: https://www.mdpi.com/article/10.3390/ijms26031162/s1.
