# Supplementary material for: Emergence and Cytogenetic Clonal Evolution of Chromosome 7 Abnormalities in Myeloid Malignancies: Investigating the Role of Telomere Dysfunction
Source: Int J Mol Sci. 2025 Jan 29;26(3):1162. doi: 10.3390/ijms26031162 (PMC11817968; doi:10.3390/ijms26031162)
Supplement: Supplementary file 1 [file ijms-26-01162-s001.zip › Supplementary Data.pdf]

Supplementary data

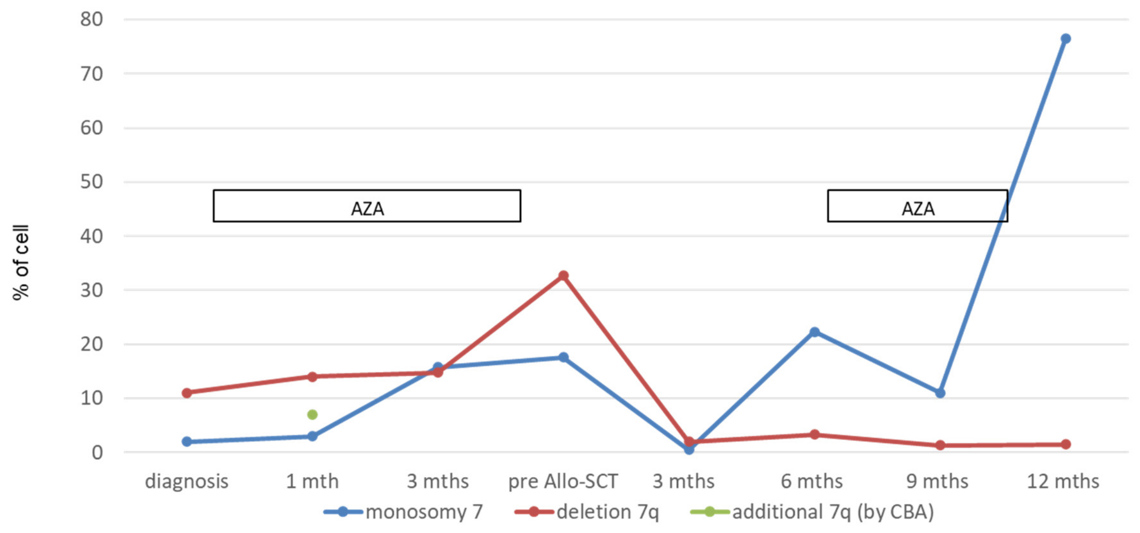

Figure S1. Graphic representing the dynamics of clones with different abn(7q) in a case with CCE7 (#ID212); AZA: Azacitidine

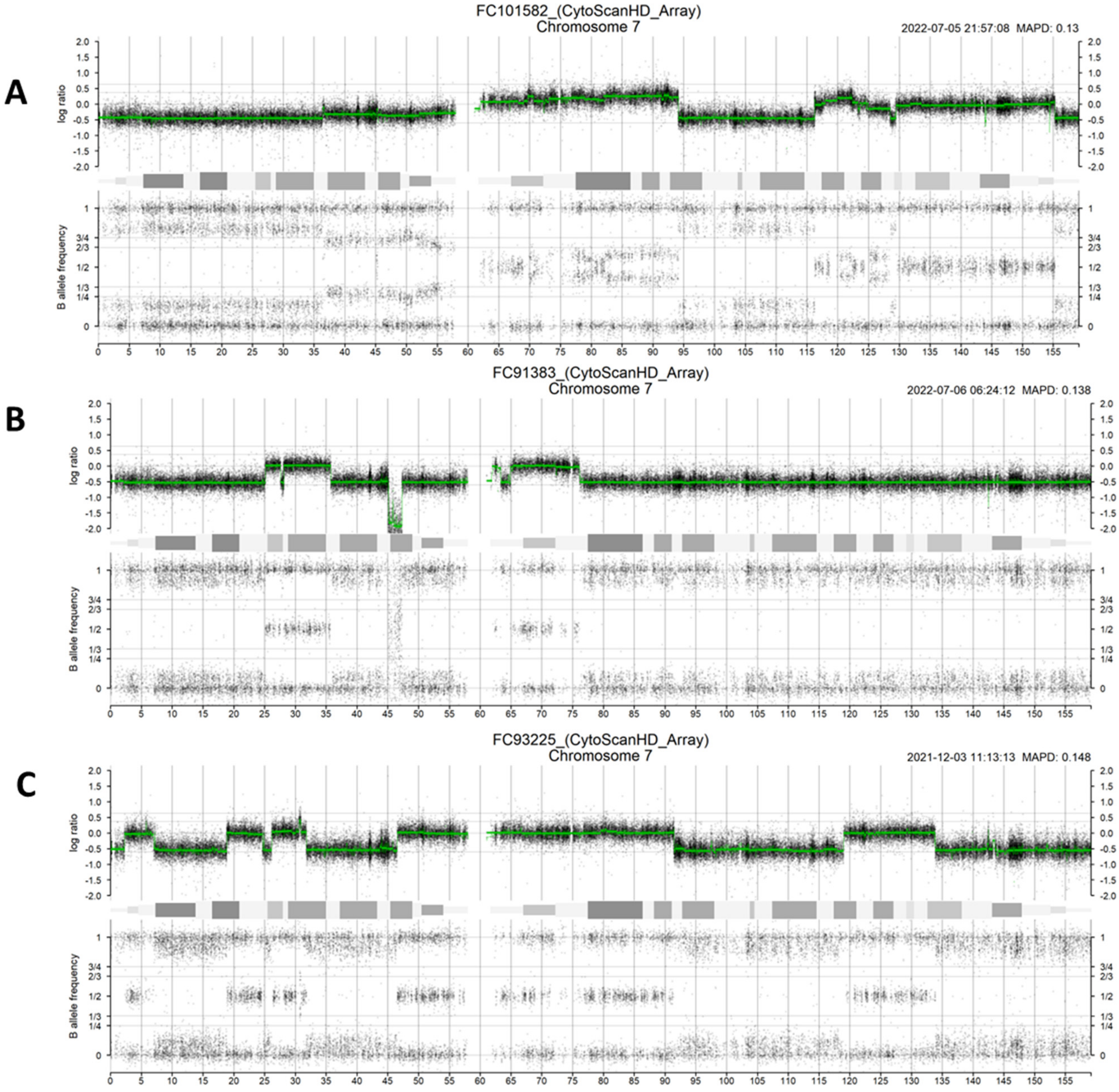

Figure S2. A) SNP array profile of chromosome 7 of ID214, showing complex mechanism in the acquisition of monosomy 7 through CCE7. B) C) SNP array profile of chromosome 7 of two cases (ID41 and ID65) with -7 by CBA revealing complex pattern of rearrangements and incomplete loss of chromosome 7.

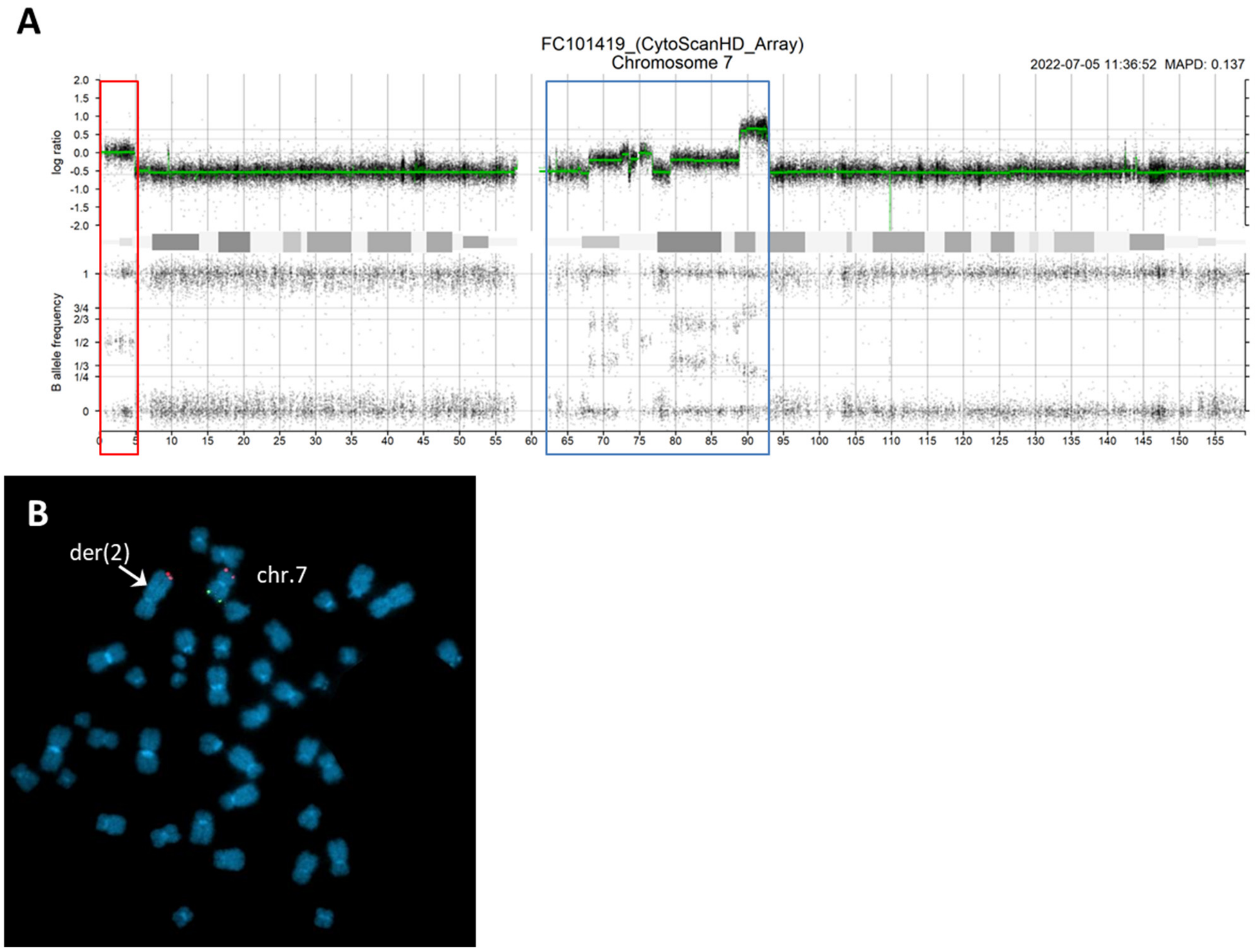

Figure S3. SNP array profiles of chromosome 7 in cases with complete monosomy 7 by CBA, revealing an incomplete loss of chromosome 7. A) Case (ID207) showing loss of the majority of chromosome 7 with the exception of two region: one 7q11-7q21 region showing complex rearrangement with multiple breakpoint and CN variations (blue box) and one 7p region (arr[GRCh38] 7p22.3p21.1(43,361_9,541,826)x2)) with a normal copy number(red box). B) FISH analysis with subtelomeric 7 probes revealing the loss of 7qter and the translocation of 7pter region on 2qter.

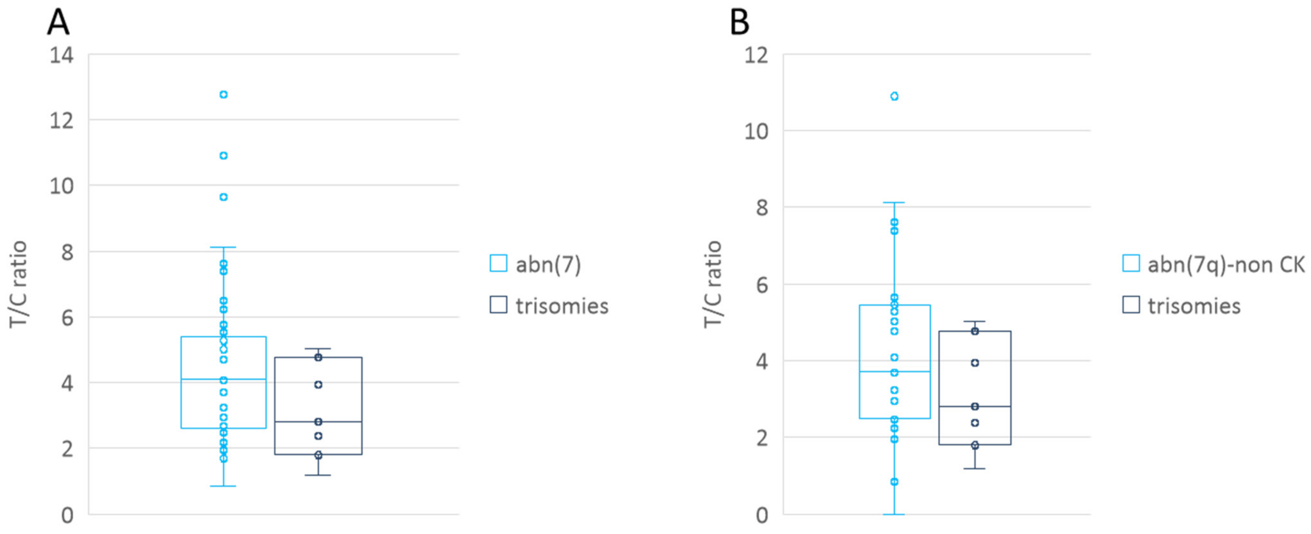

Figure S4. Telomere length analysis in patients with abn(7q) and various trisomies.

(A) Difference in average telomere length between abn(7q) and trisomies groups of patients. (B) Average telomere length between abn(7q) without complex karyotype and trisomies groups of patients.

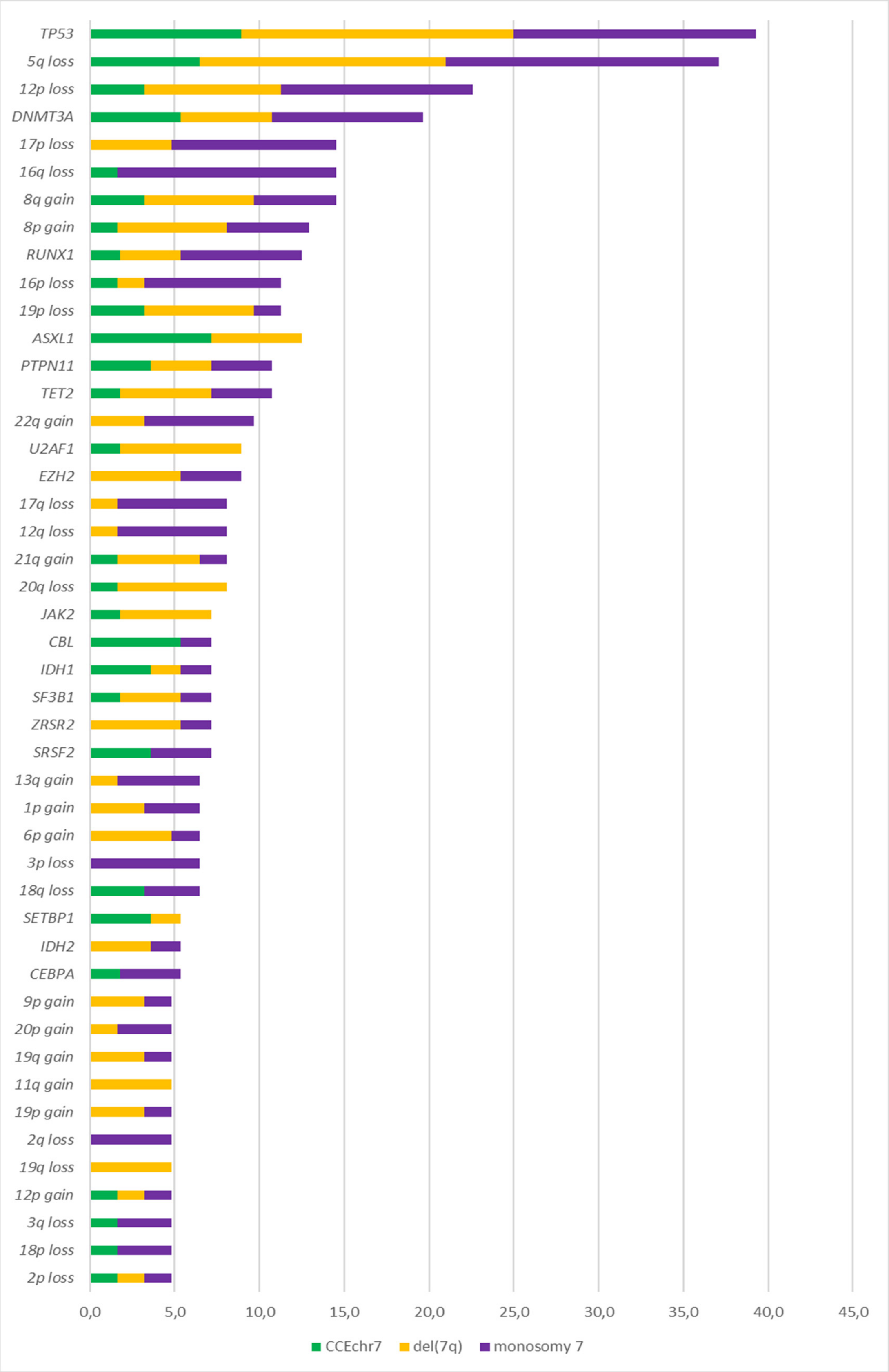

Figure S5. Co-existing somatic mutations and chromosomal aberrations in myeloid neoplasm with abn(7q). Percentage of co-associated mutation and cytogenetic abnormalities in patients with abn(7).

Table S2. Variants identified in TBD related genes

Gene variant	Type of mutation	Protein Change	Ref_Transcript	dbSNP	ACGM classification	AMP classification	ClinVAr	VAF(%)
**PARN:c.22_40del**	frameshift	p.Phe8fs	NM_002582.3	not reported	LP	VUS(tier 3)	not reported	51,3
**PARN:c.21T>C**	splice variant		NM_002582.3	rs1394666330	VUS	VUS(tier 3)	not reported	52,6
**MDM4:c.1120A>C**	missense	p.Lys374Gln	NM_002393.4	rs41299595	LB	VUS(tier 3)	LB	47,9
**PARN:c.1297C>A**	missense	p.Leu433Ile	NM_002582.3	rs201782700	VUS	VUS(tier 3)	VUS	47,1
**CTC1:c.25C>T**	missense	p.Pro9Ser	NM_025099.5	rs1034774367	VUS	VUS(tier 3)	VUS	23,7
**RTEL1:c.3392C>G**	missense	p.Thr1131Arg	NM_001283009.1	rs372852392	VUS	VUS(tier 3)	VUS	57,6
**POT1:c.1127A>G**	missense	p.Gln376Arg	NM_015450.2	rs143635917	VUS	VUS(tier 3)	conflicting	21,5
**MDM4:c.1100C>T**	missense	p.Ser367Leu	NM_002393.4	rs201947927	VUS	VUS(tier 3)	not reported	47,5
**RTEL1:c.2544_2546delTGGinsCGA**	missense	p.Gly849Asp	NM_001283009.1	not reported	VUS	VUS(tier 3)	not reported	32,4
**CTC1:c.2386-10A>G**	splice variant		NM_025099.5	rs746382653	VUS	VUS(tier 3)	VUS	100,0
**PARN:c.1785T>G**	missense	p.Asp595Glu	NM_002582.3	rs200434143	VUS	VUS(tier 3)	VUS	49,9
**MDM4:c.1162C>G**	missense	p.Pro388Ala	NM_002393.4	rs61754765	VUS	VUS(tier 3)	not reported	50,6
**TERT:c.2545A>C**	missense	p.Met849Leu	NM_198253.2	not reported	VUS	VUS(tier 3)	not reported	50,3
**WRAP53:c.305C>T**	missense	p.Thr102Ile	NM_018081.2	rs759428765	VUS	VUS(tier 3)	VUS	46,7
**BLM:c.3062A>G**	missense	p.Asn1021Ser	NM_000057.3	rs369629509	VUS	VUS(tier 3)	conflicting	48,1
**RTEL1:c.2624G>A**	missense	p.Arg875Lys	NM_001283009.1	rs200565373	VUS	VUS(tier 3)	VUS	51,4
**RTEL1:c.3592G>A**	missense	p.Glu1198Lys	NM_001283009.1	rs771457769	VUS	VUS(tier 3)	VUS	50,7
**ACD: c.22G>A**	missense	p.Val8Ile	NM_001082486.1	rs149365469	VUS	VUS(tier 3)	conflicting	47,4
**TERT:c.3184G>A**	missense	p.Ala1062Thr	NM_198253.2	rs35719940	VUS	B(tier 4)	conflicting	47,5
**TERT:c.1234C>T**	missense	p.His412Tyr	NM_198253.2	rs34094720	B	B(tier 4)	conflicting	50,2
**RTEL1:c.3354G>C**	missense	p.Met1118Ile	NM_001283009.1	not reported	VUS	VUS(tier 3)	not reported	52,0
**RTEL1:c.2544_2546delTGGinsCGA**	missense	p.Gly849Asp	NM_001283009.1	not reported	VUS	VUS(tier 3)	not reported	47,8
**WRAP53:c.668G>C**	missense	p.Gly223Ala	NM_018081.2	rs753668696	VUS	VUS(tier 3)	VUS	12,0
**MDM4:c.1120A>C**	missense	p.Lys374Gln	NM_002393.4	rs41299595	LB	VUS(tier 3)	LB	52,0
**TERT:c.3184G>A**	missense	p.Ala1062Thr	NM_198253.2	rs35719940	VUS	B(tier 4)	conflicting	51,9
**NAF1c.208G>C**	missense	p.Val70Leu	NM_001082486.1	rs746104270	VUS	VUS(tier3)	VUS	49,8
**TEN1:c.262G>A**	missense	p.Val88Met	NM_001113324.2	rs201043191	VUS	VUS(tier 3)	not reported	52,5

**TINF2:c.1213G>A**	missense	p.Glu405Lys	NM_001113324.2	not reported	VUS	VUS(tier 3)	not reported	50,1
**TINF2:c.734C>A**	missense	p.Ser245Tyr	NM_001113324.2	rs142777869	B	B(tier 4)	B/LB	49,9
**NAF1:c.1478A>C**	missense	p.Tyr493Ser	NM_001082486.1	rs143001503	VUS	VUS(tier 3)	conflicting	49,0
**POT1:c.1228G>C**	missense	p.Asp410His	NM_015450.2	rs79314063	B	B(tier 4)	conflicting	70.3
**MDM4:c.1153C>G**	missense	p.Leu385Val	NM_002393.4	not reported	VUS	VUS(tier3)	not reported	52,0
**ZCCHC8:c.1325C>T**	missense	p.Ala442Val	NM_017612.4	rs375394115	VUS	VUS(tier3)	VUS	46,0
**ACD: c.22G>A**	missense	p.Val8Ile	NM_001082486.1	rs149365469	VUS	VUS(tier 3)	conflicting	48,9
**ACD:c.243-6_243-4dup**	splice variant		NM_001082486.1	not reported	VUS	VUS(tier 3)	not reported	51,8

**Table S3. CNV in patients with different group of chromosome 7 abnormalities**							
	**All (n=62)**	**CCE7 (n=16)**	**del(7q) (n= 20)**	**-7 (n= 26)**	**Total**	**Total %**	***p value***
**5q loss**	23(37,1)	4(25,0)	9(47,4)	10(38,5)	0,0	0,0	ns
**12p loss**	14(22,6)	2(12,5)	5(26,3)	7(26,9)	0,0	0,0	ns
**8q gain**	9(14,5)	2(12,5)	4(21,1)	3(11,5)	0,0	0,0	ns
**16q loss**	**9(14,5)**	**1(6,3)**	**0**	**8(30,8)**	**0,0**	**0,0**	**p=0,008**
**17p loss**	9(14,5)	0	3(15,8)	6(23,1)	0,0	0,0	ns
**8p gain**	8(12,9)	1(6,3)	4(21,1)	3(11,5)	0,0	0,0	ns
**19p loss**	7(11,3)	2(12,5)	4(21,1)	1(3,8)	0,0	0,0	ns
**16p loss**	7(11,3)	**1(6,3)**	1(5,3)	5(19,2)	0,0	0,0	ns
**22q gain**	6(9,7)	0	2(10,5)	4(15,4)	0,0	0,0	ns
**20q loss**	**5(8,1)**	**1(6,3)**	**4(21,1)**	**0**	**0,0**	**0,0**	**p=0,094**
**21q gain**	5(8,1)	1(6,3)	3(15,8)	1(3,8)	0,0	0,0	ns
**12q loss**	5(8,1)	0	1(5,3)	4(15,4)	0,0	0,0	ns
**17q loss**	5(8,1)	0	1(5,3)	4(15,4)	0,0	0,0	ns
**18q loss**	4(6,5)	2(12,5)	0	2(7,7)	0,0	0,0	ns
**3p loss**	**4(6,5)**	**0**	**0**	**4(15,4)**	**0,0**	**0,0**	**p=0,037**
**6p gain**	4(6,5)	0	3(15,8)	1(3,8)	0,0	0,0	ns
**1p gain**	4(6,5)	0	2(10,5)	2(7,7)	0,0	0,0	ns
**13q gain**	4(6,5)	0	1(5,3)	3(11,5)	0,0	0,0	ns
**2p loss**	3(4,8)	1(6,3)	1(5,3)	1(3,8)	0,0	0,0	ns
**18p loss**	3(4,8)	1(6,3)	0	2(7,7)	0,0	0,0	ns
**3q loss**	3(4,8)	1(6,3)	0	2(7,7)	0,0	0,0	ns
**12p gain**	3(4,8)	1(6,3)	1(5,3)	1(3,8)	0,0	0,0	ns
**19q loss**	**3(4,8)**	**0**	**3(15,8)**	**0**	**0,0**	**0,0**	**p=0,044**
**2q loss**	3(4,8)	0	0	3(11,5)	0,0	0,0	ns
**19p gain**	3(4,8)	0	2(10,5)	1(3,8)	0,0	0,0	ns
**11q gain**	**3(4,8)**	**0**	**3(15,8)**	**0**	**0,0**	**0,0**	**p=0,044**
**19q gain**	3(4,8)	0	2(10,5)	1(3,8)	0,0	0,0	ns
**20p gain**	3(4,8)	0	1(5,3)	2(7,7)	0,0	0,0	ns
**9p gain**	3(4,8)	0	2(10,5)	1(3,8)	0,0	0,0	ns
**6q loss**	2(3,2)	0	1(5,3)	1(3,8)	0,0	0,0	ns

**5p loss**	2(3,2)	0	1(5,3)	1(3,8)	0,0	0,0	ns
**22q loss**	2(3,2)	0	0	2(7,7)	0,0	0,0	ns
**1q loss**	2(3,2)	0	0	2(7,7)	0,0	0,0	ns
**14q loss**	2(3,2)	0	0	2(7,7)	0,0	0,0	ns
**18p gain**	2(3,2)	0	1(5,3)	1(3,8)	0,0	0,0	ns
**6q gain**	2(3,2)	0	1(5,3)	1(3,8)	0,0	0,0	ns
**1q gain**	2(3,2)	0	2(10,5)	0	0,0	0,0	ns
**11p gain**	2(3,2)	0	2(10,5)	0	0,0	0,0	ns
**15q gain**	2(3,2)	0	0	2(7,7)	0,0	0,0	ns
**6p loss**	1(1,6)	1(6,3)	0	0	0,0	0,0	ns
**11q loss**	1(1,6)	1(6,3)	0	0	0,0	0,0	ns
**20p loss**	1(1,6)	0	1(5,3)	0	0,0	0,0	ns
**8q loss**	1(1,6)	0	0	1(3,8)	0,0	0,0	ns
**8p loss**	1(1,6)	0	0	1(3,8)	0,0	0,0	ns
**1p loss**	1(1,6)	0	0	1(3,8)	0,0	0,0	ns
**11p loss**	1(1,6)	0	0	1(3,8)	0,0	0,0	ns
**20q gain**	1(1,6)	0	0	1(3,8)	0,0	0,0	ns
**5p gain**	1(1,6)	0	0	1(3,8)	0,0	0,0	ns
**14q gain**	1(1,6)	0	0	1(3,8)	0,0	0,0	ns
**4p gain**	1(1,6)	0	1(5,3)	0	0,0	0,0	ns
**9q gain**	1(1,6)	0	0	1(3,8)	0,0	0,0	ns
**10p gain**	1(1,6)	0	0	1(3,8)	0,0	0,0	ns
**10q gain**	1(1,6)	0	0	1(3,8)	0,0	0,0	ns

Table S4. Mutations in patients with CK abn(7q)

	All (n=25)	CCE7 (n=5)	del(7q) (n=10)	-7 (n=10)
TP53	20(80)	5(100)	7(70)	8(80)
DNMT3A	4(15,4)	1(20)	1(10)	2(20)
TET2	2(7,7)	0	1(10)	1(10)
RUNX1	2(7,7)	1(20)	0	1(10)
U2AF1	2(7,7)	0	2(20)	0
ZRSR2	2(7,7)	0	1(10)	1(10)
CEBPA	2(7,7)	1(20)	0	1(10)
EZH2	1(3,8)	0	1(10)	0
SF3B1	1(3,8)	0	0	1(10)
CBL	1(3,8)	1(20)	0	0
IDH1	1(3,8)	0	1(10)	0
SRSF2	1(3,8)	0	0	1(10)
CALR	1(3,8)	1(20)	0	0
KRAS	1(3,8)	0	0	1(10)

Table S5. Mutations in patients with non-CK abn(7q)

	All (n=31)	CCE7 (n=10)	del(7q) (n=10)	-7 (n=11)
DNMT3A	7(26,6)	2(20)	2(20)	3(27,3)
ASXL1	7(26,6)	4(40)	3(30)	0
PTPN11	6(19,4%)	2(20)	2(20)	2(18,2)
RUNX1	5(16,1)	0	2(20)	3(27,3)
TET2	4(12,9%)	1(10)	2(20)	1(9,1)
EZH2	4(12,9%)	0	2(20)	2(18,2)
JAK2	4(12,9%)	1(10)	3(30)	0
U2AF1	3(9,7%)	1(10)	2(20)	0
SF3B1	3(9,7%)	1(10)	2(20)	0
SETBP1	3(9,7%)	2(20)	1(10)	0
CBL	3(9,7%)	2(20)	0	1(9,1)
IDH1	3(9,7%)	2(20)	0	1(9,1)
SRSF2	3(9,7%)	2(20)	0	1(9,1)
IDH2	3(9,7%)	0	2(20)	1(9,1)
TP53	2(6,5%)	0	2(20)	0
ZRSR2	2(6,5%)	0	2(20)	0
ETV6	2(6,5%)	2(20)	0	0
CALR	1(3,2%)	0	1(10)	0
KRAS	1(3,2%)	1(10)	0	0
CSF3R	1(3,2%)	1(10)	0	0
FLT3	1(3,2%)	1(10)	0	0
WT1	1(3,2%)	1(10)	0	0
CEBPA	1(3,2%)	0	0	1(9,1)
MPL	1(3,2%)	0	0	1(9,1)
NPM1	1(3,2%)	0	0	1(9,1)
NRAS	1(3,2%)	0	0	1(9,1)
